# Dosimetric impact of aperture‐restricted VMAT on heart dose in breast cancer treated with regional nodal irradiation

**DOI:** 10.1002/acm2.70775

**Published:** 2026-08-26

**Authors:** Anh Hong Duong, Mandeep Kaur, Sunil W. Dutta, Tsegawbizu Gebru, Jacob Adams, Mark Cicchino, Reshma Jagsi, Sheela Hanasoge, Oluwatosin Kayode, Eduard Schreibmann, Hania Al‐Hallaq, Matthew Thomas, Xiaofeng Yang, Shadab Momin, Justin Roper, Kirk Luca, Jie Ding

**Affiliations:** ^1^ Department of Radiation Oncology Emory University Atlanta Georgia USA; ^2^ Medical Dosimetry Program Southern Illinois University Carbondale Illinois USA; ^3^ Department of Radiation and Cellular Oncology University of Chicago Chicago Illinois USA

**Keywords:** aperture‐restricted, breast VMAT, heart sparing, regional nodal irradiation

## Abstract

**Background:**

Volumetric modulated arc therapy (VMAT) is increasingly used for breast and chest wall (CW) treatment with regional nodal irradiation (RNI) because of its ability to sculpt dose around complex target volumes. However, VMAT can increase low‐dose radiation to surrounding normal tissues and particularly increase cardiac dose.

**Purpose:**

To evaluate the dosimetric impact of a strategic aperture‐restricted beam geometry, compared with traditional open‐field geometry in VMAT planning for comprehensive left‐sided breast/CW irradiation with RNI across three linac platforms. The impact of convergence mode settings was also investigated.

**Methods:**

Fourteen patients with left‐sided breast/CW irradiation including comprehensive RNI were retrospectively included. For each patient, a patient‐specific optimization template with a prescription of 40.05 Gy in 15 fractions was applied across three linac platforms (Varian TrueBeam, Varian Halcyon, and Elekta Versa HD), using two beam geometries: an open‐field approach, in which field sizes fully encompassed the targets, and an aperture‐restricted approach, in which the deep field border was limited to reduce beam entry to the heart and ipsilateral lung. Jaw tracking was enabled on the TrueBeam and Versa HD linacs. Each plan was also evaluated across three convergence modes (“Off,” “On,” and “Extended”), resulting in 18 plans per patient. All plans were normalized such that 95% of the breast/CW planning target volume received 95% of the prescription dose. Dosimetric endpoints include mean heart dose, ipsilateral lung V17Gy, contralateral lung V4Gy, and contralateral breast/CW V5Gy. Total monitor units (MUs) were evaluated, and patient‐specific quality assurance (PSQA) was performed for the three highest‐MU plans on each linac platform. Paired tests were used to compare across plans.

**Results:**

Target coverage was maintained after plan normalization. Aperture‐restricted VMAT significantly reduced mean heart dose compared with open‐field geometry across all linac platforms and convergence modes. The proportion of plans exceeding mean heart dose of 4 Gy was substantially lower with aperture‐restricted geometry compared to open‐field plans (2.4% vs 26.2%). The largest reduction in mean heart dose was observed with the “Off” convergence mode, decreasing from 4.20 to 2.71 Gy on TrueBeam, 3.32 to 2.58 Gy on Halcyon, and 3.97 to 2.84 Gy on Versa HD. Ipsilateral lung V17Gy was also significantly reduced with aperture‐restricted beam geometry, with only one exception on Halcyon using the “Extended” mode. No significant differences were observed in contralateral lung V4Gy, although contralateral breast/CW V5Gy increased significantly, representing a dosimetric tradeoff associated with the aperture‐restricted geometry. Aperture‐restricted beam geometry significantly increased MUs; however, PSQA passed for the three highest‐MU plans on each linac platform according to our clinical criteria. Additionally, more extensive convergence modes were associated with improved plan quality, particularly in reducing hotspot dose (D0.1cc) and enhancing cardiac and ipsilateral lung sparing.

**Conclusions:**

Aperture‐restricted beam geometry significantly reduced mean heart dose and ipsilateral lung dose while maintaining target coverage across all three linac platforms, supporting its potential clinical value in VMAT planning for comprehensive left‐sided breast/CW irradiation with RNI, albeit with increased contralateral breast/CW dose. When combined with a more extensive convergence mode, overall plan quality was further improved.

## 1 INTRODUCTION

Adjuvant radiotherapy is an essential component of breast cancer management, but for patients with left‐sided disease, incidental heart irradiation remains a clinically meaningful source of late morbidity.^1^ In a landmark population‐based study, Darby et al. demonstrated a linear association between mean heart dose and major coronary events, with an approximate 7.4% relative increase per Gy and no clear threshold for a safe dose.^1^ Recent prospective data from patients treated with modern breast radiotherapy techniques further suggest that higher cardiac dose is associated with measurable declines in cardiac function during intermediate follow‐up, which may contribute to future cardiovascular risks.^2^ Collectively, these studies reinforce the importance of minimizing heart dose in breast radiotherapy.

Comprehensive irradiation of the whole breast or chest wall (CW), including the ipsilateral regional nodal irradiation (RNI), is routinely indicated for selected patients with high‐risk breast cancer, and many patients with node‐positive disease receive RNI as suggested by observational studies,^3^, ^4^ impacting tens of thousands of patients each year in the United States alone. RNI typically includes the axillary, supraclavicular (SCV), and internal mammary (IMN) nodal basins, along with the post‐mastectomy CW in patients receiving mastectomy or the intact breast in patients receiving breast‐conserving surgery. Randomized trials and observational studies have demonstrated that comprehensive nodal irradiation improves locoregional control and clinical outcomes in appropriately selected patients, with the Early Breast Cancer Trialists Collaborative Group meta‐analysis suggesting an absolute reduction in breast cancer mortality of 2.7% even among patients with involvement of only 1–3 lymph nodes.^4^, ^5^ However, the inclusion of RNI increases target complexity, and IMN irradiation results in higher dose closer to the heart, further challenging cardiac sparing. Deep‐inspiration breath‐hold (DIBH), a technique that reduces cardiac dose by increasing the separation between the heart and CW, has been widely adopted in breast radiotherapy.^6^ Nevertheless, minimizing the heart dose during the planning stage remains critical.

Volumetric modulated arc therapy (VMAT), an advanced intensity‐modulated radiation therapy technique, has been increasingly adopted in breast and CW radiotherapy with RNI because it improves high dose conformity, homogeneity, and target coverage for complex geometries, including nodal volumes.^7^ At the same time, the rotational delivery and reliance on the multileaf collimator (MLC) for complex shaping may increase the volume of normal tissues receiving low‐dose radiation (“low‐dose bath”), including the heart, compared with conventional tangential or hybrid techniques.^7^ Even with DIBH, the low‐dose bath can be challenging to control due to several factors including arc geometry, optimization strategy, and machine dependent leakage and scatter. Multiple VMAT planning approaches have been proposed to mitigate cardiac dose, including restricted or tangential arc designs, split‐arc techniques, and off‐isocentric planning.^7^, ^8^, ^9^, ^10^, ^11^ More recently, attention has shifted toward collimation and field width effects. In the context of early‐stage whole breast partial arc VMAT, Guo et al. reported that increased jaw‐defined field width increased low‐dose cardiac exposure, whereas overly aggressive jaw restriction could compromise target coverage.^12^ Given this tradeoff, it remains uncertain whether an appropriately applied aperture‐restricted strategy can provide consistent, clinically meaningful cardiac sparing without compromising target coverage for comprehensive irradiation, including RNI, and whether the dosimetric benefit translates across linac platforms with different collimation architectures. Further, convergence mode optimization has shown promise for reducing dose to healthy tissues beneath a concave shaped target in total scalp irradiation,^13^ yet its role is unclear in comprehensive breast/CW planning.

In this study, we performed a multiplatform evaluation of aperture‐restricted beam geometry for cardiac sparing in comprehensive left‐sided breast/CW irradiation with RNI across three linac configurations (Varian TrueBeam, Varian Halcyon, and Elekta Versa HD). These platforms have unique field shaping capabilities using either the MLC alone or the MLC in combination with one or two collimating jaws. Using a platform appropriate asymmetric collimation to truncate the aperture on the cardiac side of the field, we compared aperture‐restricted and standard open‐field VMAT geometries to determine whether aperture restriction yields consistent reduction in cardiac dose without compromising target coverage for anatomically challenging comprehensive breast/CW radiotherapy. The beam geometry techniques were investigated in combination with convergence mode settings to explore the effects on cardiac sparing and overall plan quality.

## 2 METHODS

### 2.1 Patient selection

With institutional review board approval for this retrospective study, fourteen patients were selected who received VMAT radiotherapy to the left‐sided breast/CW and regional nodes between 2022 and 2025. All patients were treated with DIBH. The median patient age was 60 years (range, 29–77). All patients had either confirmed nodal involvement or clinically suspected/prior nodal involvement. Among the 14 patients, 9 were post‐mastectomy, including 5 with tissue expanders; of these 9 post‐mastectomy patients, 6 had undergone bilateral mastectomy. The breast/CW volumes ranged from 284 to 1859 cc.

### 2.2 Target volumes and organs at risk (OARs)

Clinical target volumes (CTVs) were contoured by a radiation oncologist and included the breast or CW, first three intercostal spaces of the internal mammary chain, axillary nodal levels I–III, and SCV lymph nodes. Planning target volumes (PTVs) were generated according to the Imaging and Radiation Oncology Core nodal contouring atlas.^14^ Breast/CW PTVs were created using a 5 mm three‐dimensional expansion from the corresponding CTV, excluding the heart and limiting extension across the midline. IMN PTVs were expanded by 5 mm medially, laterally, superiorly, and inferiorly, excluding sternum, lung, heart, and breast/CW. Axillary nodal PTVs were expanded isotropically by 5 mm with exclusion of lung tissue. SCV nodal PTVs were expanded by 5 mm in all directions except medially, with exclusion of the thyroid, trachea, esophagus, lung, vertebral body, and a 5 mm margin from the skin surface.

### 2.3 Patient‐specific optimization templates

**FIGURE 1:**
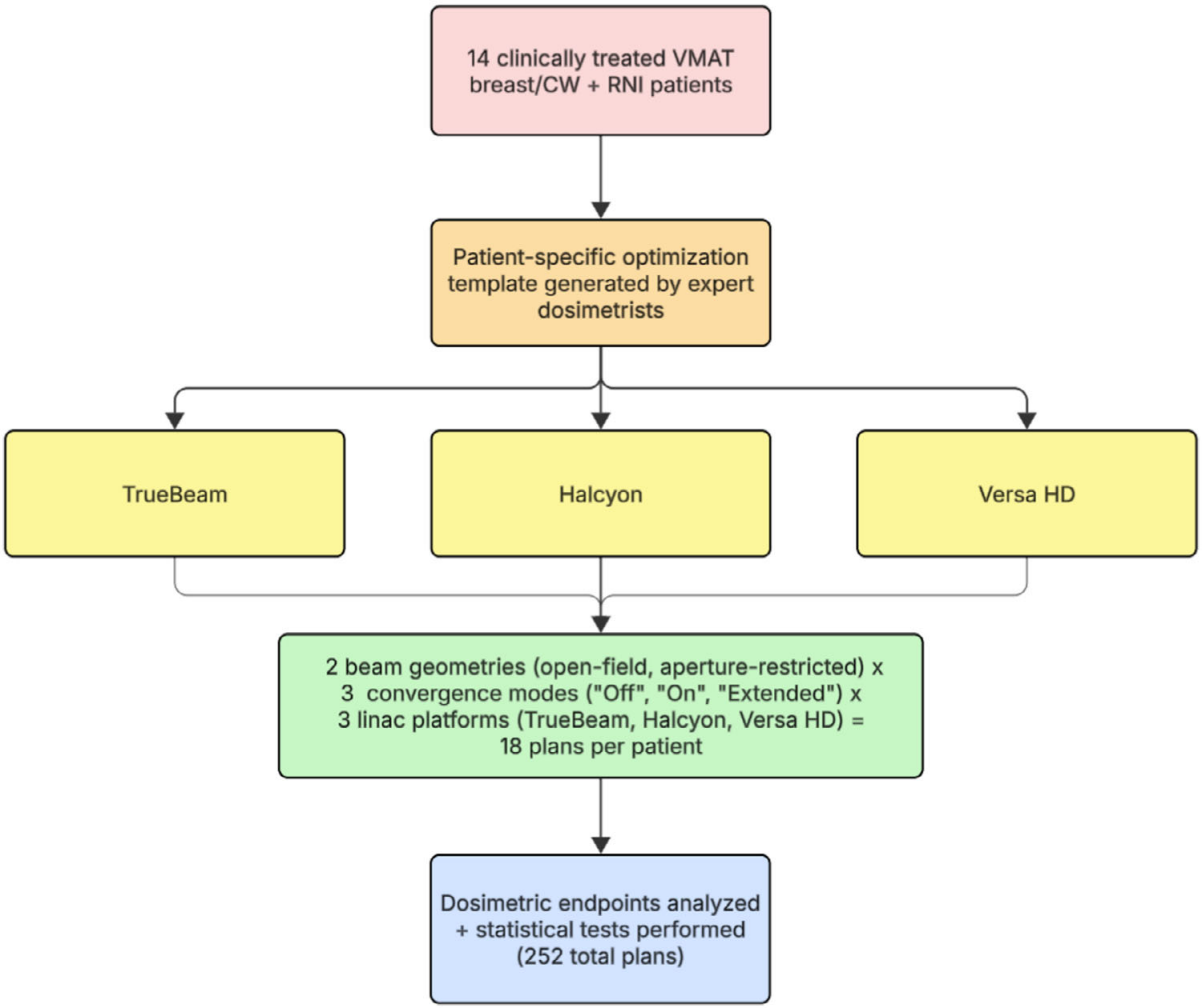
Fourteen previously treated VMAT breast/CW + RNI patients were selected, and expert dosimetrists developed patient‐specific optimization templates using the ASTRO–ASCO–SSO guideline as a planning framework 3. Each case was then reoptimized without user interaction on three delivery platforms (Varian TrueBeam, Varian Halcyon, and Elekta Versa HD) using both open‐field and aperture‐restricted geometries across three convergence modes (“Off”, “On”, “Extended”), yielding 18 plans per patient (252 total plans). Dosimetric endpoints were subsequently extracted and analyzed.

An initial plan was generated for each patient by expert dosimetrists using Eclipse V16.1 treatment planning system (Varian Medical Systems). All plans were created using 6‐MV flattening filter‐free (6X‐FFF) photon beams with a prescribed dose of 40.05 Gy delivered in 15 fractions. Dose was calculated using the analytic anisotropic algorithm (AAA). Plans were iteratively optimized using Photon Optimizer V16.1 toward dosimetric objectives informed by the PMRT clinical practice guideline developed by the American Society for Radiation Oncology (ASTRO), American Society of Clinical Oncology (ASCO), and Society of Surgical Oncology (SSO), hereafter referred to as the ASTRO–ASCO–SSO guideline.^3^ While the expert dosimetrists aimed to generate balanced, high‐quality plans, not all planning goals were achieved during development of the patient‐specific optimization templates. The use of these templates was to maintain consistent optimization conditions across all plans for the same patient, thereby enabling a controlled evaluation of the dosimetric effect from beam geometry design and convergence mode settings. The final optimization objectives were saved as a patient‐specific template and then applied to 18 plans for each patient. These 18 plans varied by the combination of the treatment delivery platform with different collimator configurations (Varian TrueBeam, Varian Halcyon, Elekta Versa HD), beam geometry (open‐field and aperture‐restricted), and convergence mode setting (“Off”, “On”, “Extended”). Figure 1 shows the workflow of this study.

### 2.4 Linac characteristics and collimator configurations

Three linac platforms were used in this study: Varian TrueBeam, Varian Halcyon, and Elekta Versa HD. Varian TrueBeam is a C‐arm linac equipped with two conventional collimator jaws and a tertiary MLC system mounted on movable carriages below the jaws. Varian Halcyon is an O‐ring linac featuring a dual‐layer MLC system without conventional jaws. Elekta Versa HD is a C‐arm linac equipped with conventional lower jaws and an MLC system relacing the upper jaws.Table S1 summarizes the details of characteristics and collimator configurations of the three linac platforms. The BEVs of an example control point for each of the three machines are shown in Figure S1.

### 2.5 Field geometry

#### 2.5.1 Open‐field geometry

**FIGURE 2:**
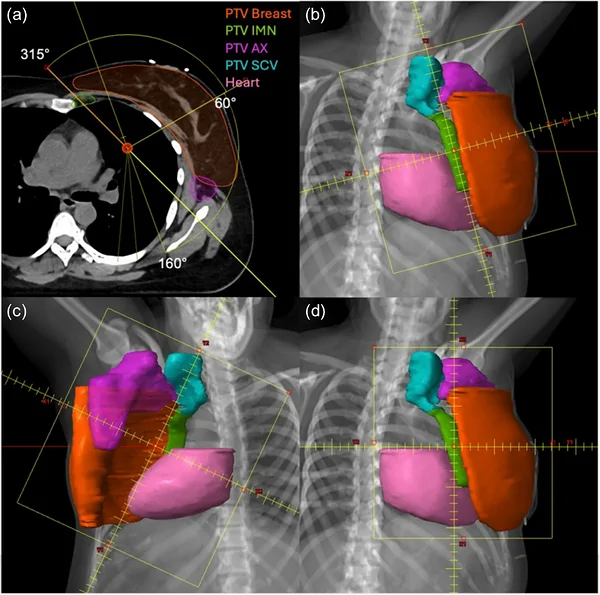
Open‐field geometry for a representative case on a Varian TrueBeam linac. Field size fully encompassed the PTVs and jaw tracking was enabled. (a) Isocenter placement and partial‐arc VMAT geometry. The isocenter was positioned along the tangential line of sight. Partial arcs were used from gantry 315° to 60° and 60° to 160°. (b) BEV for Arc 1 at gantry 315° with collimator 20°. (c) BEV for Arc 3 at gantry 160° with collimator 335°. (d) BEV for Arc 5 at gantry 315° with collimator 90°. Contour legends are shown in (a).

Open‐field beam geometry was used as the reference configuration, with a representative example on Varian TrueBeam shown in Figure 2. The isocenter was positioned near the midpoint of the superior–inferior extent of the target volumes and shifted posteriorly along the tangential beam path into the ipsilateral lung (Figure 2a). Five partial arcs were designed, with individualized arc start and stop angles based on patient anatomy and target extent, typically spanning angular ranges of approximately 308° to 55° and 55° to 165° (averaged ranges; exact angles varied slightly depending on patient anatomy). Arcs were split at gantry of 50°– 60° because, as the gantry approaches this range, the beam becomes increasingly en face to the breast/CW, and the BEV projection of the target and heart change substantially thereby challenging the ability of the MLC to achieve both target coverage and cardiac sparing. Therefore, the arc was intentionally split to allow for adjustment (flipping) of the collimator angle to maintain a field edge in the X‐direction predominantly parallel to the CW, as shown in Figure 2b and c. As a result, the MLC leaves travel along the short axis of the CW (see Figure S1), which is favorable for dose sculpting. This approach is consistent with a previously described split‐arc VMAT strategy for breast and nodal irradiation.^8^

Collimator angles for the first four partial arcs were selected to align with breast/CW geometry and facilitate nodal coverage, as shown in Figure 2b and c. A fifth partial arc with 90° collimator angle, shown in Figure 2d, was used to improve dose conformity and provide additional control points in cases where beam entry was limited by the arm or chin. In these plans, all fields were allowed to fully encompass the target volumes with jaw tracking enabled on TrueBeam and Versa HD. Halcyon is a jawless treatment platform and therefore does not use conventional jaw tracking. Table S2 summarizes the beam geometries used for all patients in this study.

#### 2.5.2 Aperture‐restricted geometry

**FIGURE 3:**
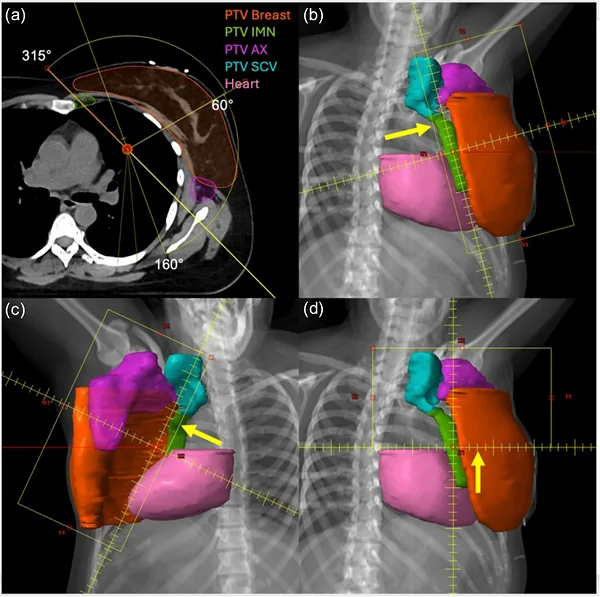
Aperture‐restricted geometry for a representative case on a Varian TrueBeam linac. Beam geometry was identical to Figure 2 with the exception for truncation of the deep field border. The yellow arrows indicate the restricted field setting by jaws on TrueBeam (a) Isocenter placement and partial‐arc VMAT geometry. The isocenter was positioned along the tangential line of sight. Partial arcs were used from gantry 315° to 60° and 60° to 160°. (b) BEV for Arc 1 at gantry 315° with collimator 20°. (c) BEV for Arc 3 at gantry 160° with collimator 335°. (d) BEV for Arc 5 at gantry 315° with collimator 90°. Contour legends are shown in (a).

Arc geometry was identical in the open‐field and aperture‐restricted plans; the only difference was the field size in the X direction along the deep field border. Aperture restriction was implemented by selectively retracting the X‐jaws or limiting the MLCs defining the deep tangential field border toward the isocenter by approximately 1–2 cm on arcs 1–4 to create partial half‐beam blocking to limit cardiac exposure. Figure 3 illustrates the aperture‐restricted beam geometry used in this study. For TrueBeam, which is equipped with X‐jaws upstream of the tertiary MLCs, aperture restriction was achieved by setting an X‐jaw to define the deep tangential field border. For Halcyon and Versa HD which do not use secondary jaws upstream of the MLCs, MLC leaves were limited within the optimizer (using the “limit leaves” feature in Eclipse) to achieve similar limited field widths. The fifth partial arc was restricted to achieve superior target coverage, as shown in Figure 3d, creating a half‐beam block on the cardiac side to prevent beam divergence into the heart. Jaw tracking was enabled on TrueBeam and Versa HD.

### 2.6 Optimization and dose calculation

VMAT plans were generated using patient‐specific optimization templates, and once applied, each plan was optimized without user interaction. Flash was created using a virtual bolus with a thickness of 1.0 cm and a density of −500 Hounsfield units applied over the breast/CW PTV.^15^ A flash PTV was generated by expanding the clinical PTV anteriorly and laterally by 1 cm each to encourage MLC extension beyond the patient's external surface (as shown in Figure S2). The virtual bolus was linked to each treatment field prior to optimization. During optimization, this structure is treated as a target and assigned a low to moderate priority to generate sufficient cost to drive MLC opening beyond the patient's surface. This approach for achieving skin flash is intended to ensure adequate surface dose coverage and improve plan robustness against setup uncertainty, respiratory motion, and daily anatomical changes such as breast swelling or deformation.^15^ After plan optimization, the virtual bolus was removed from each treatment field, and the plans were recalculated to generate the final dose distribution.

The dosimetric impact of convergence mode was investigated by optimizing each plan using three settings (“Off”, “On”, and “Extended”). Convergence mode is a feature in Eclipse that modifies the maximum number of iterations and the criteria for cost function convergence. Compared with the “Off” mode, the “On” and “Extended” modes allow additional iterations and stricter convergence criteria, which enables more extensive optimization to potentially improve plan quality, with the trade‐off of increased optimization time.^13^, ^16^, ^17^, ^18^

AAA was used for intermediate and final dose calculation with a dose grid of 2.5×2.5 ×2.5 mm^3^. All plans were normalized to ensure that 95% of the prescribed dose covered 95% of the breast/CW PTV.

### 2.7 Dosimetric analysis

Plan evaluation was performed on dosimetric endpoints in accordance with the ASTRO–ASCO–SSO guideline.^3^ Although this study was not intended to generate treatment plans that meet all dosimetric constraints specified in the ASTRO–ASCO–SSO guideline, the recommended constraints were used as reference criteria for plan quality evaluation to facilitate dosimetric comparisons.

As breast/CW PTV coverage was standardized by normalization, target coverage evaluation was focused on the nodal PTVs, with V95% and V90% assessed for the axillary, SCV, and IMN. Hotspots were quantified using D0.1cc for the breast/CW PTV and each nodal PTV. OAR evaluation was primarily focused on the heart and left lung, including heart mean dose and left lung V17Gy. Right breast/CW V5Gy and right lung V4Gy were also evaluated.

Comparisons between the open‐field geometry and the proposed aperture‐restricted geometry were performed using the paired Wilcoxon signed‐rank test separately for each linac platform and convergence mode, with *p <* 0.05 considered statistically significant. The effect of convergence mode (“Off”, “On”, and “Extended”) was analyzed using the Friedman test, with Holm correction applied to account for multiple comparisons among the three convergence modes. Holm‐adjusted *p <* 0.05 was considered statistically significant.

Total MUs were recorded for all plans, with patient‐specific quality assurance (QA) performed for the three plans with the highest total MUs on each treatment platform to evaluate plan deliverability and dosimetric accuracy. Measurements were acquired using electronic portal imaging devices (EPIDs) field‐by‐field for TrueBeam and Halcyon, and MapCHECK3 (Sun Nuclear, Melbourne, FL, USA) for Versa HD. Gamma passing rates were calculated using 3% dose difference and 2 mm distance‐to‐agreement criteria with a 10% dose threshold and global normalization.

## 3 RESULTS

### 3.1 Comparison of representative dose distributions

**FIGURE 4:**
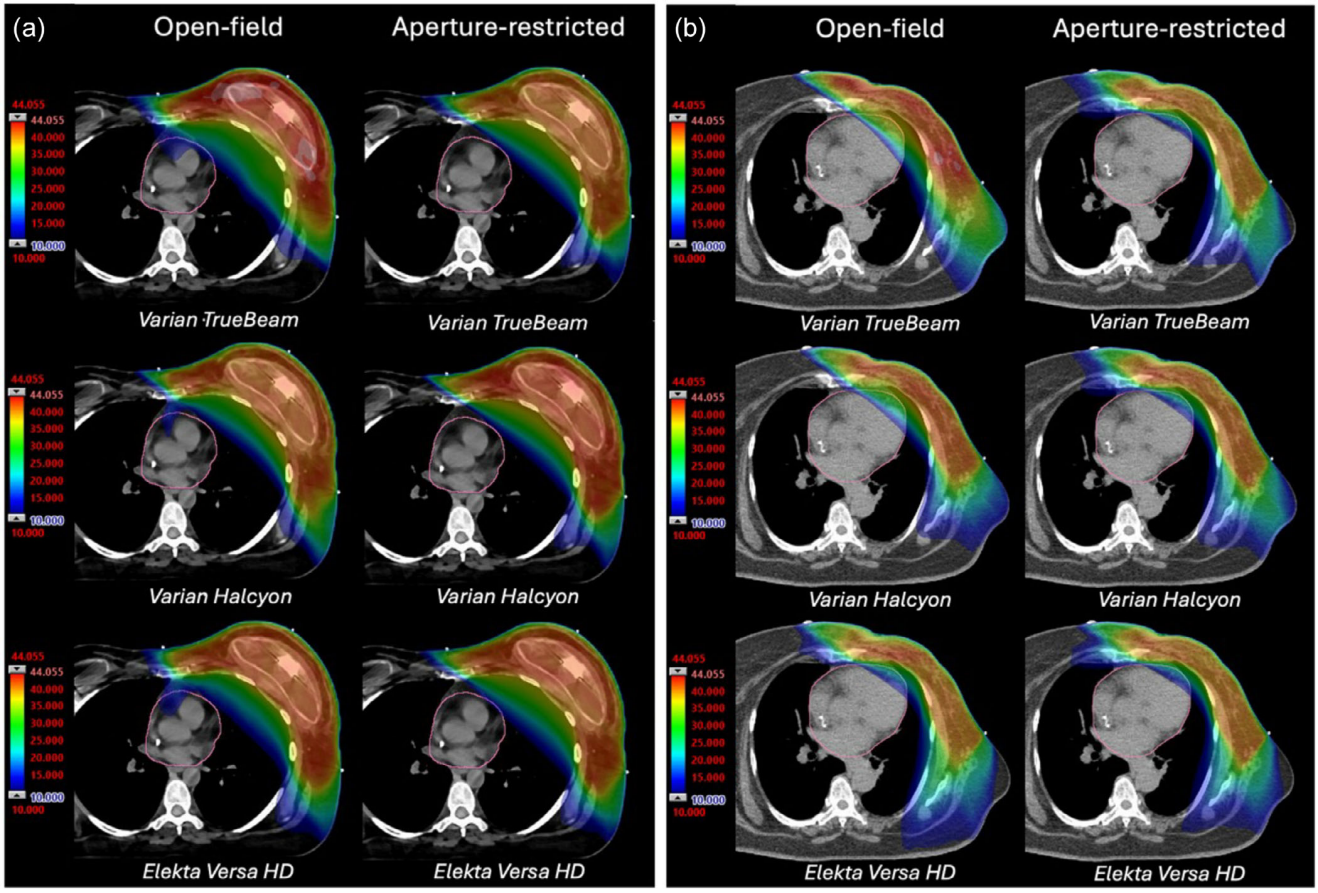
Representative dose distributions for (a) a left‐sided tissue expander and (b) a CW irradiation case comparing open‐field and aperture‐restricted VMAT plans, optimized with the “Extended” convergence mode. Aperture restriction maintains target coverage while reducing low‐dose spread to the heart and left lung.

Representative dose distributions comparing open‐field and aperture‐restricted plans are shown in Figure 4 (a tissue expander case and a CW case). In both examples, aperture‐restricted geometry limited the beam aperture closest to the heart, resulting in reduced low‐dose spread into the heart while maintaining coverage of the breast/CW and regional nodal targets.

### 3.2 Target coverage and hotspots

Target dose was evaluated for all PTVs, using V95%, V90% and D0.1cc. A comprehensive summary of mean values and standard deviations across all patients is provided in Table S3 for each treatment platform, field geometry, and convergence mode.

A summary of selected results is presented. Since all plans were normalized to achieve breast/CW PTV V95% receiving 95% prescription dose, adequate breast/CW coverage was maintained across all plans. The acceptable hotspot constraint for breast/CW PTV (D0.1cc ≤ 115%) was met in 104 of 126 open‐field plans and 116 of 126 aperture‐restricted plans. For nodal targets, the acceptable coverage criterion of V90% ≥ 90% was achieved in 124/126 open‐field plans and 126/126 aperture‐restricted plans for SCV PTV, 125/126 and 126/126 plans for axillary PTV, and 105/126 and 106/126 plans for IMN PTV, respectively.

Aperture‐restricted field geometry was associated with statistically significant dosimetric improvements, particularly for axillary PTV (p‐values provided in Table S4). The axillary PTV coverage improvement was observed on all three treatment platforms, and hotspot reduction was notable on TrueBeam. For example, on TrueBeam plans optimized with the “Off” convergence mode, the mean axillary PTV V95% increased from 92.56% to 97.34% (*p =* 0.001), V90% increased from 97.78% to 99.49% (*p <* 0.001), and D0.1cc decreased from 113.94% to 110.15% (*p =* 0.009) when aperture‐restricted fields were used instead of the open fields.

Regarding convergence mode settings, no consistent impact on target coverage was observed overall, whereas significant hotspot reductions were noted (p‐values provided in Table S5). Although a few statistically significant differences were identified for nodal coverage, these findings were not uniform across platforms or beam geometry designs. In particular, a more extensive convergence mode was associated with slightly reduced coverage of the SCV and axillary PTVs in some open‐field plans, whereas modest improvements in IMN PTV coverage were observed in certain aperture‐restricted plans. In contrast, “On” and “Extended” convergence modes were consistently associated with significant hotspot reductions compared with the “Off” setting, with the “Extended” mode providing additional improvement over the “On” mode in most scenarios.

### 3.3 Mean heart dose

Mean heart dose was consistently lower with aperture‐restricted plans compared with open‐field plans across all linac platforms and convergence modes as shown in Table 1, with Holm‐adjusted comparisons demonstrating statistically significant reductions in heart mean heart dose (all *p <* 0.01). The largest reduction in mean heart dose was observed when comparing aperture‐restricted geometry with open‐field geometry optimized using the “Off” convergence mode, decreasing from 4.20 to 2.71 Gy on TrueBeam, 3.32 to 2.58 Gy on Halcyon, and 3.97 to 2.84 Gy on Versa HD (all *p <* 0.001).

**TABLE 1:** Dosimetric comparison of open‐field and aperture‐restricted VMAT plans across linac platforms and convergence modes for heart mean dose (Dmean), left lung V17Gy, right lung V4Gy, right breast/CW V5Gy, and total MUs. Values are presented as mean ± standard deviation. P‐values represent Wilcoxon signed‐rank paired comparisons between open‐field and aperture‐restricted plans within each machine and convergence mode. NS = not statistically significant; MU = monitor unit.

Machine	Convergence mode	Field geometry	Heart Dmean (Gy)	*p*	Left lung V17Gy (%)	*p*	Right lung V4Gy (%)	*P*	Right breast/CW V5Gy (%)	*p*	MUs	*p*
TrueBeam	Off	Open‐field	4.20 ± 2.50	<0.001	31.17 ± 11.18	<0.001	19.45 ± 14.99	0.715 (NS)	5.00 ± 4.65	0.002	712 ± 112	<0.001
		Aperture‐restricted	2.71 ± 0.85		23.12 ± 5.09		18.57 ± 11.12		8.95 ± 7.34		1034 ± 128	
	On	Open‐field	3.54 ± 1.95	<0.001	26.35 ± 9.30	<0.001	14.69 ± 14.24	0.761(NS)	5.86 ± 5.64	0.042	740 ± 128	<0.001
		Aperture‐restricted	2.49 ± 0.81		21.44 ± 4.95		14.55 ± 11.91		8.36 ± 7.78		1055 ± 145	
	Extended	Open‐field	3.26 ± 1.72	<0.001	24.92 ± 9.34	0.002	14.25 ± 16.79	0.626 (NS)	5.70 ± 6.12	0.463 (NS)	769 ± 147	<0.001
		Aperture‐restricted	2.34 ± 0.74		20.41 ± 4.77		12.84 ± 12.57		6.26 ± 5.54		1124 ± 180	
Halcyon	Off	Open‐field	3.32 ± 1.54	<0.001	26.69 ± 9.41	0.001	19.84 ± 14.47	0.855 (NS)	4.11 ± 4.80	<0.001	826 ± 110	<0.001
		Aperture‐restricted	2.58 ± 0.83		22.26 ± 5.27		19.06 ± 10.28		8.69 ± 7.31		1101 ± 122	
	On	Open‐field	2.84 ± 1.18	<0.001	22.59 ± 6.19	0.001	16.37 ± 13.06	0.808 (NS)	4.67 ± 6.23	<0.001	854 ± 119	<0.001
		Aperture‐restricted	2.41 ± 0.76		20.75 ± 5.15		16.49 ± 11.06		8.85 ± 8.44		1096 ± 135	
	Extended	Open‐field	2.43 ± 0.87	0.007	20.41 ± 5.66	0.153 (NS)	13.70 ± 14.30	0.463 (NS)	4.48 ± 4.57	0.017	971.0 ± 203	<0.001
		Aperture‐restricted	2.19 ± 0.64		19.69 ± 5.08		14.14 ± 11.99		6.40 ± 5.84		1208 ± 224	
Versa HD	Off	Open‐field	3.97 ± 1.95	<0.001	28.86 ± 8.68	0.001	22.45 ± 17.06	0.542 (NS)	4.31 ± 4.92	<0.001	804 ± 112	<0.001
		Aperture‐restricted	2.84 ± 0.90		23.51 ± 5.37		19.40 ± 9.87		11.62 ± 10.32		1093 ± 141	
	On	Open‐field	3.38 ± 1.47	0.001	25.07 ± 7.32	0.001	19.19 ± 15.88	0.808 (NS)	4.49 ± 5.35	<0.001	860 ± 129	<0.001
		Aperture‐restricted	2.68 ± 0.79		21.93 ± 5.22		19.18 ± 11.33		11.01 ± 9.97		1142 ± 151	
	Extended	Open‐field	3.20 ± 1.32	0.001	23.63 ± 6.39	0.001	17.42 ± 15.47	1.000 (NS)	4.83 ± 6.48	<0.001	873 ± 139	<0.001
		Aperture‐restricted	2.58 ± 0.72		21.13 ± 5.33		17.20 ± 11.25		9.96 ± 9.28		1147 ± 150	

**FIGURE 5:**
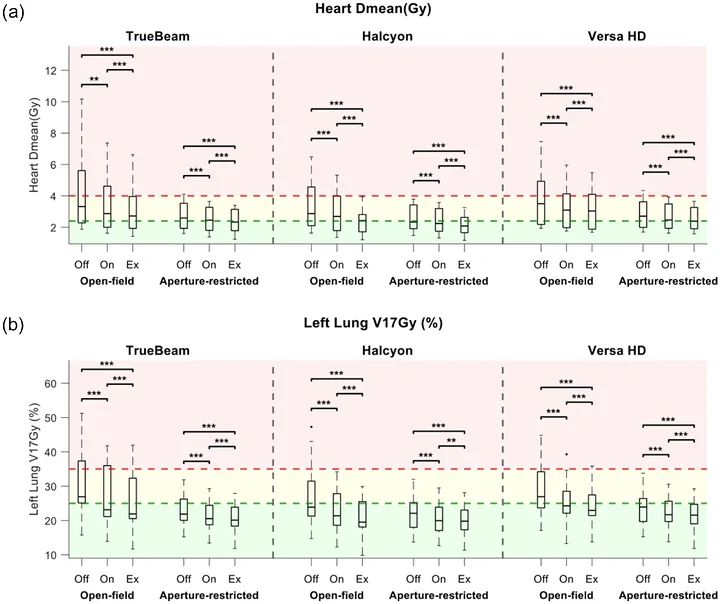
Distribution of mean heart dose (Dmean) (a) and left lung V17Gy (b) across the three treatment platforms (TrueBeam, Halcyon, and VersaHD), comparing two field geometries (open‐field and aperture‐restricted), each optimized using three convergence modes (“Off”, “On” and “Ex”, where “Ex” denotes the “Extended” convergence mode). The green dashed line (2.4 Gy) indicates the ideal mean heart dose goal, and the red dashed line (4 Gy) represents the acceptable deviation per guideline. Statistically significant differences between convergence modes are also indicated (*p < 0.05, **p < 0.01, ***p < 0.001).

Figure 5a illustrates the distribution of mean heart dose across the three treatment platforms, comparing open‐field plans and aperture‐restricted plans optimized using three convergence modes. Among the 126 open‐field plans evaluated, 33 plans (26.2%) exceeded the acceptable mean heart dose threshold of 4 Gy. In comparison, only 3 of the 126 (2.4%) aperture‐restricted plans exceeded this limit, and all three were optimized with the “Off” convergence mode. All aperture‐restricted plans achieved mean heart dose < 4 Gy when optimized using “On” and “Extended” convergence modes, indicating consistent improvement in cardiac dose sparing across all three treatment platforms. In addition, a greater proportion of aperture‐restricted plans achieved the more stringent ideal mean heart dose goal of 2.4 Gy as observed in Figure 5a, particularly when “Extended” convergence mode was used. The most favorable heart sparing results were observed for aperture‐restricted plans optimized with “Extended” mode on the Halcyon platform, which achieved an averaged mean heart dose of 2.19 Gy.

Figure 5a also demonstrates significant differences among convergence modes, showing that “On” and “Extended” convergence modes resulted in significantly lower mean heart dose compared with the “Off” mode, and that the “Extended” mode provided the greatest reduction. Detailed p‐values for all statistical comparisons on convergence modes are reported in Table S6.

### 3.4 Left lung

For left lung, the intermediate‐dose metric V17Gy was significantly reduced with aperture‐restricted plans across treatment platforms and convergence modes, with the exception of Halcyon using the “Extended” convergence mode (Table 1). For example, on TrueBeam with the “Off” convergence mode, the mean V17Gy across all patients decreased from 31.17% to 23.12% (*p <* 0.001) when aperture‐restricted fields were used instead of open‐field geometry.

The distribution of V17Gy across linac platforms and convergence modes is illustrated in Figure 5b. Among the 126 open‐field plans evaluated, 19 plans (15.1%) did not meet the acceptable V17Gy goal of 35%, and 55 plans (43.7%) exceeded the more stringent ideal goal of 25%. In comparison, all 126 aperture‐restricted plans met the acceptable goal, whereas 34 plans (27.0%) did not meet the ideal goal. Figure 5b also demonstrates significant differences among convergence modes, showing that optimization with the “On” and “Extended” convergence modes consistently reduced left lung V17Gy compared with the “Off” mode, with “Extended” mode providing the greatest improvement. Detailed p‐values for the statistical comparisons on convergence modes are reported in Table S6.

### 3.5 Right lung and right breast/CW

Dosimetric evaluation was also performed for contralateral structures using right lung V4Gy and right breast/CW V5Gy, as summarized in Table 1.

For right lung V4Gy, no significant differences were observed between aperture‐restricted and open‐field geometries across treatment platforms (all p > 0.05; Table 1). For convergence modes, optimizing using the “On” and “Extended” settings was generally associated with lower right lung V4Gy values compared with the “Off” mode, with the “Extended” mode often providing additional reduction (Table S6).

For the right breast/CW, aperture‐restricted plans resulted in significantly higher V5Gy values than open‐field plans (Table 1), except for TrueBeam plans optimized using the “Extended” convergence mode. The effect of convergence mode on right breast/CW dose was limited overall, although the use of the “Extended” mode was associated with statistically significant reductions in V5Gy for aperture‐restricted plans on TrueBeam and Halcyon (Table S6).

Additional dosimetric metrics, including contralateral breast/CW Dmean and Dmax, as well as metrics corresponding to the additional suggested OAR dose considerations specified in the ASTRO–ASCO–SSO guideline, are summarized in Table S7.

### 3.6 MU, patient‐specific QA, and optimization time

Aperture‐restricted plans consistently demonstrated significantly higher MUs compared with open‐field plans across all three linac platforms (Table 1), indicating increased modulation and plan complexity. The use of “Extended” convergence mode further increased the plan MUs particularly for TrueBeam and Halcyon (Table S6).

Patient‐specific QA was performed for the three plans with the highest MUs on each linac platform following our clinical workflow. The average gamma passing rates were 98.93% and 99.87% for TrueBeam and Halcyon (measured using EPIDs, averaged per field), and 98.93% for Versa HD (measured using MapCHECK3, averaged per plan). All gamma passing rates were ≥ 95% except for one field on TrueBeam that achieved 93.7%, which remained above the action limit of 90%. The QA results indicate that despite increased modulation and plan complexity, the aperture‐restricted beam geometry did not compromise plan deliverability or dosimetric accuracy.

The optimization time using different convergence modes was recorded. Based on our testing, which was performed using patient‐specific optimization templates without manual intervention during optimization, the approximate planning time was 50 minutes when using the “Extended” mode, compared to 18 minutes for “On” and 6 minutes for “Off”.

Additional dosimetric metrics, including the contralateral breast/CW Dmean and Dmax and the additional OAR dose considerations specified in the ASTRO–ASCO–SSO guideline, are reported in Table S7.

## 4 DISCUSSION

The primary aim of this study was to investigate the dosimetric impact of the aperture‐restricted beam geometry in VMAT planning for comprehensive left‐sided breast/CW irradiation with RNI, particularly in reducing mean heart dose. Across the three linac platforms evaluated, the aperture‐restricted geometry consistently resulted in significant reductions in mean heart dose compared with conventional open‐field VMAT plans (all *p <* 0.01), while maintaining adequate target coverage for both breast/CW and nodal volumes. The greatest heart sparing was generally observed when plans were optimized using the most stringent convergence setting, “Extended” mode (with “On” mode also demonstrating improved performance compared with the “Off” mode). The reduction in heart dose is primarily due to the truncation of the deep tangential beam border throughout arc delivery, which limits beam entry through the heart and minimizes its low‐dose bath. When comparing across beam geometries and convergence modes, a ∼2 Gy reduction in mean heart dose (averaged across all patients) was observed on TrueBeam with aperture‐restricted geometry using the “Extended” convergence mode (2.34 Gy), relative to open‐field geometry with the “Off” mode (4.20 Gy). Based on Darby et al.,^1^ which reported that the rate of major coronary events increases by approximately 7.4% per Gy of mean heart dose, this magnitude of reduction in mean heart dose may translate to a clinically meaningful reduction in cardiac toxicity. In addition to cardiac sparing, the aperture‐restricted approach was also associated with significant reductions in ipsilateral lung dose when assessed using the clinically relevant V17Gy metric. Similarly, a ∼10% reduction in averaged left lung V17Gy was observed on TrueBeam with aperture‐restricted geometry using the “Extended” convergence mode (20.41%), relative to open‐field geometry with the “Off” mode (31.17%), which may be associated with a reduction in radiation‐induced lung toxicity based on prior studies investigating the effects of lung dose–volume constraints on pulmonary functions and radiological changes.^19^ Overall, these findings indicate that the proposed aperture‐restricted beam geometry can improve dose sparing to both the heart and the left lung, while maintaining target coverage, across all three treatment platforms with varying collimation systems, supporting its potential clinical value in VMAT planning for left‐sided breast/CW irradiation with RNI.

The dosimetric impact of field geometry on contralateral OARs was examined using right lung V4Gy and right breast/CW V5Gy. While no significant differences were observed in right lung V4Gy, aperture‐restricted plans resulted in a statistically significant increase in right breast/CW V5Gy. This increase in contralateral breast dose is applicable primarily to patients with an intact contralateral breast, as opposed to those who underwent bilateral mastectomy. One possible explanation is that restricting the deep field apertures limits the available beam directions for covering the medial portion of the PTV. To maintain target coverage, a greater dose contribution may be required from the remaining beam directions, including left‐to‐right directions, which may produce additional exit dose through the contralateral breast (see Figure S3). A similar tradeoff between improved heart sparing and increased contralateral breast dose has also been reported in previous studies, such as the split‐arc VMAT technique that improved heart and ipsilateral lung sparing at the expense of increased contralateral breast dose.^8^ Nevertheless, the proposed aperture‐restricted geometry provides a practical planning strategy when further reduction of heart and ipsilateral lung dose is clinically desired, particularly in patients who have undergone bilateral mastectomy for whom the risk‐benefit tradeoff is likely favorable. Additional plan optimization may also be performed in clinical practice to mitigate contralateral breast dose, depending on specific planning priorities.

In addition to beam geometry, this study also evaluated the influence of convergence mode settings on plan quality. The convergence mode feature allows adjustment of the maximum number of optimization iterations and convergence criteria, with the “Extended” mode representing the most stringent setting by permitting additional iterations and tighter convergence requirements. Previous studies have suggested that more extensive convergence settings improve dosimetric outcomes across multiple treatment sites,^13^, ^16^, ^17^, ^18^ at the cost of longer optimization times. However, the role of convergence settings in VMAT planning for comprehensive breast/CW irradiation with RNI has not been previously investigated. One study has evaluated convergence modes for breast/CW irradiation with axillary nodes and reported that the “Extended” mode failed to achieve acceptable dose distributions, so this mode was excluded from their evaluation.^16^ This observation is likely related to the use of the older Eclipse version 15.6 in that study, in which a limitation affected the performance of the “Extended” mode. In contrast, the present study using Eclipse version 16.1 demonstrated that the “Extended” mode functioned as intended and was associated with improved plan quality, particularly in reducing hotspots as well as heart and ipsilateral lung dose for comprehensive breast/CW irradiation with RNI across all three linac platforms. However, the optimization time required for the “Extended” mode can be longer as reported in Results. This trade‐off between potential dosimetric benefits and prolonged planning time should be considered when selecting convergence settings in clinical practice. We also observed that with the “Extended” convergence mode, dosimetric differences between open‐field and aperture‐restricted plans were reduced, with some metrics becoming comparable (this trend is seen in Figure 5 and Table 1; for example, no significant difference was found for left lung V17 on Halcyon when using “Extended” mode). This is likely due to the increased optimization iterations and tighter convergence criteria, allowing better plan quality from improved optimization and reducing the dosimetric impact of beam geometry. These findings suggest that for less challenging cases with adequate separation between the heart and CW, if the conventional open‐field geometry is used (e.g., due to its simpler implementation and lower MUs), the “Extended” convergence mode is preferred to improve plan quality.

A major strength of this study is that the investigation was performed across three linac platforms with different collimation configurations. By evaluating the dosimetric impact of aperture‐restricted beam geometry and convergence modes across all three systems, this study provides a systematic cross‐platform assessment that has not previously been reported for VMAT breast planning, particularly in the context of comprehensive breast/CW irradiation with RNI. Previous VMAT breast studies have focused primarily on early‐stage whole‐breast irradiation or on treatment involving limited nodal volumes.^7^, ^8^, ^9^, ^10^, ^11^, ^12^ In contrast, the present study specifically investigated comprehensive breast/CW irradiation with comprehensive RNI, which represents a more anatomically complex planning geometry and poses greater challenges for achieving high plan quality.

Despite the findings presented, several limitations should be acknowledged. This study was conducted on a relatively small cohort of 14 patients. Additional statistical analyzes of the mean heart dose reduction demonstrated statistical power of 88.2%–89.5%, and the corresponding 95% confidence intervals remained entirely above zero across all convergence modes (Table S8), providing additional support for the observed reduction in mean heart dose with the aperture‐restricted geometry. In addition, plan evaluation was based on a limited set of dosimetric goals. For example, cardiac sparing was assessed using mean heart dose alone, without evaluation of cardiac substructures such as the left anterior descending artery and left ventricle, which have been shown to be strongly associated with radiation‐induced cardiac risk in recent studies.^20^ Future work should include a more comprehensive investigation on the dosimetric impact of aperture‐restricted geometry, including cardiac substructures, in a larger patient cohort. In addition, all plans were generated using patient‐specific optimization templates to ensure that differences among the resulting plans could be attributed to beam geometry or convergence mode rather than to differences in optimization objectives or planner intervention. This approach does not fully reflect a standard clinical planning workflow. Unlike routine clinical planning, the resulting plans were not further refined through additional optimization runs or objective adjustments and therefore may not reflect the best achievable plan quality. Although the expert dosimetrists aimed to produce high‐quality plans in accordance with the ASTRO–ASCO–SSO guideline, there were no strict constraints on beam geometry or planning strategies used when generating the patient‐specific optimization templates, and individual planning approaches also varied based on experience and preference. However, the purpose of the patient‐specific templates was to maintain consistent optimization objectives across all plans for the same patient, thereby allowing fair comparisons of the dosimetric effects of beam geometry and convergence modes. Likewise, the gantry start/stop angles and collimator angles were customized for each patient using a split‐arc technique, as described in the Methods and summarized in Table S2, but were intentionally kept identical between the aperture‐restricted and open‐field plans for the same patient to isolate the dosimetric impact of the aperture‐restriction technique. Furthermore, the proposed aperture‐restricted beam geometry significantly increased the total MUs compared with the open‐field plans, which indicates increased plan complexity, likely because of the smaller effective field sizes and greater MLC modulation. However, the three highest‐MU plans on each treatment platform all passed patient‐specific QA according to our clinical standards, suggesting that the aperture‐restricted technique remains clinically deliverable with acceptable dosimetric accuracy. The increased MU may lead to longer delivery times for aperture‐restricted plans, particularly on Halcyon, where the relatively low maximum dose rate (800 MU/min) can result in gantry speed reduction when the dose rate limit is reached during VMAT delivery. Delivery times between open‐field and aperture‐restricted plans were comparable on TrueBeam and Versa HD due to their higher maximum dose rates for 6X‐FFF (1400 and 2200 MU/min, respectively). Adding a fifth arc, while beneficial for target coverage, increases treatment delivery time and requires additional breath‐hold for the patient; however, the split‐arc technique (arcs 1–4) may offer a practical advantage by requiring shorter beam‐on time per arc, which may be more feasible for breath‐hold delivery.^8^

## 5 CONCLUSION

This study demonstrates that aperture‐restricted beam geometry significantly reduces mean heart dose and ipsilateral lung dose while preserving target coverage in VMAT planning for breast/CW irradiation with RNI. Additionally, the use of more extensive convergence modes was associated with improved plan quality, particularly in reducing hotspot dose and improving cardiac sparing. Prospective clinical implementation testing and evaluation are warranted to validate these findings further.

## AUTHOR CONTRIBUTIONS

**Anh Hong Duong**: Investigation; methodology; writing—original Draft. **Mandeep Kaur**: Writing—review & editing. **Sunil W. Dutta**: Writing—review & editing. **Tsegawbizu Gebru**: Investigation; writing—review & editing. **Jacob Adams**: Investigation; writing—review & editing. **Mark Cicchino**: Investigation; writing—review & editing. **Reshma Jagsi**: Writing—review & editing. **Sheela Hanasoge**: Writing—review & editing. **Oluwatosin Kayode**: Writing—review & editing. **Eduard Schreibmann**: Writing—review & editing. **Hania Al‐Hallaq**: Writing—review & editing. **Matthew Thomas**: Data curation; writing—review & editing. **Xiaofeng Yang**: Writing—review & editing. **Shadab Momin**: Investigation; writing—review & editing. **Justin Roper**: Methodology; writing—review & editing. **Kirk Luca**: Supervision; conceptualization; investigation; methodology; validation; visualization; writing—original draft; writing—review & editing. **Jie Ding**: Supervision; conceptualization; investigation; methodology; validation; visualization; formal analysis; writing—original draft; writing—review & editing.

## CONFLICT OF INTEREST STATEMENT

Reshma Jagsi has received personal fees from the Greenwall Foundation, Doris Duke Charitable Foundation, the National Institutes of Health, the Blue Cross Blue Shield Association, Physicians Education Resource, Bioascend, the American Medical Association, Hawks Quindel Law, and Mintz Levin Law; and grants paid to her institution from the National Institutes of Health, the Doris Duke Charitable Foundation, the American Cancer Society, the Susan G. Komen Foundation, and PreludeDX.

## ETHICS STATEMENT

This retrospective study was approved by the Institutional Review Board (IRB) of Emory University.

## Supporting information



Supporting Information: acm270775‐sup‐0001‐Figure S1‐S3.docx

Supporting Information: acm270775‐sup‐0002‐Table S1‐S8.docx

## Data Availability

The data underlying this study are subject to institutional and regulatory restrictions and are therefore not publicly available. De‐identified data may be made available from the corresponding authors upon reasonable request and with appropriate institutional approvals, including a Data Use Agreement, according to the institutional policy.
