# Supplementary material for: Dosimetric impact of aperture‐restricted VMAT on heart dose in breast cancer treated with regional nodal irradiation
Source: J Appl Clin Med Phys. 2026 Aug 26;27(9):e70775. doi: 10.1002/acm2.70775 (PMC13507928; doi:10.1002/acm2.70775)
Supplement: Supplementary file 1 [file ACM2-27-e70775-s001.docx]

**Table S1.** Linac Characteristics, including the collimator and MLC configurations for Varian TrueBeam, Varian Halcyon, and Elekta Versa HD.

|  | **Varian TrueBeam** | **Varian Halcyon** | **Elekta Versa HD** |
| --- | --- | --- | --- |
| Linac type | C-arm | O-ring | C-arm |
| Maximum gantry speed | 6°/s | 24°/s | 6°/s (5.5°/s in our practice) |
| Jaws | Conventional jaws in both X and Y directions | Jawless | Conventional lower jaws (Y direction only) |
| MLC systems | Millennium 120 MLC (60 leaf pairs), on movable carriages below the jaws | Stacked and staggered dual-layer MLCs (29 proximal + 28 distal pairs) | Agility 160 MLC (80 leaf pairs), replacing upper jaws |
| Leaf width | 5 mm (40 central pairs), 10 mm (20 peripheral pairs) | 10 mm (effective 5 mm due to staggered design) | 5 mm |
| Maximum field size | 40 × 40 cm² (static) 40 × 32 cm² (VMAT) | 28 × 28 cm² | 40 × 40 cm² |
| Maximum leaf travel | 15 cm beyond carriage | 28 cm | 32.5 cm (12.5 cm across central axis) |
| Maximum leaf speed | 2.5 cm/s | 5 cm/s | 6.5 cm/s |
| Maximum dose rate (6X-FFF) | 1400 MU/min | 800 MU/min | 2200 MU/min |

* All reported dimensions (cm, cm^2^) are defined at the isocenter plane.

**Table S2.** Gantry and collimator angle ranges for all treatment plans.

| **Arc** | **Gantry Start (°)** | **Gantry End (°)** | **Collimator Angle (°)** |
| --- | --- | --- | --- |
| 1 | 300 - 315 | 50 - 60 | 9 - 25 |
| 2 | 50 - 60 | 160 - 170 | 335 - 350 |
| 3 | 160 - 170 | 50 - 60 | 332 - 355 |
| 4 | 50 - 60 | 300 - 315 | 7 - 28 |
| 5 | 300 - 315 | 50 - 60 | 90 |

**Table S3.** Mean ± standard deviation of PTV coverage metrics (V95%, V90%, D0.1cc) for the breast/CW and nodal targets for each treatment platform, field geometry, and convergence mode. Plans were normalized such that 95% of the breast/CW PTV received 95% of the prescription dose. Bold values indicate the better result between open-field and aperture-restricted geometries within each convergence mode for each linac platform (higher values for V95% and V90%, and lower values for D0.1cc). CW = chest wall; SCV = supraclavicular nodes; AX = axillary nodes; IMN = internal mammary nodes.

| Machine | Convergence mode | Field geometry | PTV_breast/CW | | PTV_SCV | | | PTV_AX | | | PTV_IMN | | |
| --- | --- | --- | --- | --- | --- | --- | --- | --- | --- | --- | --- | --- | --- |
|  |  |  | V90% (%) | D0.1cc (%) | V95%(%) | V90%(%) | D0.1cc(%) | V95%(%) | V90%(%) | D0.1cc(%) | V95%(%) | V90%(%) | D0.1cc(%) |
| TrueBeam | Off | Open-field | 98.48 ± 0.61 | 114.53 ± 4.35 | 93.82 ± 6.76 | 98.25 ± 3.12 | 112.53 ± 4.71 | 92.56 ± 6.38 | 97.78 ± 3.20 | 113.94 ± 4.62 | **86.92 ± 12.58** | 94.03 ± 10.11 | 110.83 ± 3.02 |
|  |  | Aperture-restricted | **98.69 ± 0.51** | **112.38 ± 2.85** | **95.74 ± 3.70** | **98.81 ± 2.43** | **110.26 ± 2.83** | **97.34 ± 2.05** | **99.49 ± 0.61** | **110.15 ± 2.41** | 84.49 ± 8.30 | **94.16 ± 5.29** | **109.72 ± 2.67** |
|  | On | Open-field | 98.78 ± 0.59 | 111.92 ± 5.43 | 91.93 ± 7.83 | 97.51 ± 4.25 | 110.34 ± 5.79 | 91.16 ± 7.12 | 97.64 ± 2.75 | 111.36 ± 4.95 | 84.67 ± 13.87 | 94.39 ± 10.07 | 108.52 ± 3.40 |
|  |  | Aperture-restricted | **98.91 ± 0.43** | **110.45 ± 3.00** | **95.67 ± 2.67** | **99.08 ± 1.10** | **108.87 ± 2.86** | **96.70 ± 2.18** | **99.50 ± 0.47** | **108.55 ± 2.51** | **84.94 ± 8.04** | **95.43 ± 4.61** | **108.02 ± 2.95** |
|  | Extended | Open-field | 98.94 ± 0.57 | 111.42 ± 4.77 | 93.30 ± 5.86 | 98.46 ± 2.14 | 110.18 ± 5.21 | 90.33 ± 7.09 | 97.60 ± 2.50 | 110.97 ± 4.76 | **86.06 ± 12.99** | 95.27 ± 9.47 | 107.52 ± 3.46 |
|  |  | Aperture-restricted | **99.04 ± 0.41** | **109.60 ± 2.75** | **96.17 ± 2.57** | **99.33 ± 0.60** | **107.94 ± 2.65** | **96.29 ± 3.21** | **99.59 ± 0.33** | **107.83 ± 2.33** | 84.80 ± 10.86 | **95.77 ± 4.64** | **107.22 ± 2.93** |
| Halcyon | Off | Open-field | 98.60 ± 0.73 | **112.09 ± 4.87** | **94.59 ± 4.08** | **98.70 ± 1.36** | 110.28 ± 5.19 | 92.44 ± 7.30 | 98.33 ± 1.95 | 110.94 ± 4.50 | **82.20 ± 14.84** | 92.40 ± 9.88 | **109.13 ± 2.09** |
|  |  | Aperture-restricted | **98.63 ± 0.59** | 112.28 ± 3.55 | 94.25 ± 4.54 | 98.40 ± 2.45 | **109.20 ± 2.34** | **96.78 ± 3.68** | **99.51 ± 0.54** | **109.29 ± 2.33** | 82.05 ± 7.98 | **93.50 ± 4.99** | 109.26 ± 2.51 |
|  | On | Open-field | **98.90 ± 0.58** | **109.64 ± 3.22** | 94.88 ± 3.78 | **99.08 ± 0.93** | **107.42 ± 2.62** | 90.90 ± 8.09 | 98.16 ± 1.80 | 108.24 ± 2.51 | 82.44 ± 13.18 | 93.69 ± 8.90 | **107.27 ± 2.52** |
|  |  | Aperture-restricted | 98.84 ± 0.57 | 110.89 ± 3.82 | **94.90 ± 3.66** | 98.78 ± 1.45 | 108.18 ± 2.59 | **96.45 ± 3.46** | **99.54 ± 0.46** | **108.04 ± 2.29** | **83.13 ± 8.45** | **95.03 ± 4.94** | 107.88 ± 2.71 |
|  | Extended | Open-field | **99.15 ± 0.46** | **108.20 ± 3.37** | 94.49 ± 3.64 | 99.24 ± 0.59 | **106.10 ± 2.67** | 88.19 ± 10.05 | 97.97 ± 2.09 | **106.59 ± 2.38** | **83.83 ± 11.89** | **96.04 ± 4.54** | **105.80 ± 2.47** |
|  |  | Aperture-restricted | 99.11 ± 0.41 | 109.15 ± 2.69 | **95.65 ± 3.66** | **99.31 ± 0.66** | 107.38 ± 2.74 | **95.59 ± 4.87** | **99.57 ± 0.51** | 107.13 ± 2.26 | 83.28 ± 9.59 | 96.03 ± 3.29 | 106.52 ± 2.24 |
| Versa HD | Off | Open-field | 98.35 ± 0.68 | **112.21 ± 3.55** | **97.09 ± 3.23** | **99.50 ± 0.81** | 110.77 ± 4.03 | 95.74 ± 3.72 | 99.03 ± 1.06 | 111.15 ± 3.58 | **84.58 ± 12.52** | 92.96 ± 10.05 | 110.61 ± 3.16 |
|  |  | Aperture-restricted | **98.51 ± 0.50** | 112.53 ± 2.49 | 96.95 ± 2.88 | 99.37 ± 0.96 | **110.55 ± 2.83** | **98.27 ± 1.76** | **99.67 ± 0.41** | **110.61 ± 2.57** | 83.32 ± 8.53 | **93.17 ± 5.45** | **110.31 ± 2.48** |
|  | On | Open-field | 98.56 ± 0.67 | **110.22 ± 3.64** | **97.27 ± 2.77** | **99.63 ± 0.53** | **108.85 ± 3.02** | 95.27 ± 4.13 | 99.03 ± 1.02 | 109.29 ± 3.15 | 84.85 ± 13.00 | 93.53 ± 10.91 | **108.61 ± 2.20** |
|  |  | Aperture-restricted | **98.72 ± 0.47** | 111.48 ± 2.53 | 97.24 ± 2.27 | 99.46 ± 0.85 | 109.11 ± 2.34 | **98.36 ± 1.63** | **99.72 ± 0.35** | **109.10 ± 2.12** | **85.56 ± 7.85** | **95.44 ± 4.53** | 109.05 ± 2.26 |
|  | Extended | Open-field | 98.65 ± 0.69 | **109.44 ± 2.91** | **97.06 ± 3.01** | **99.63 ± 0.50** | **108.05 ± 2.85** | 94.83 ± 4.32 | 99.02 ± 1.02 | 108.56 ± 2.81 | 85.34 ± 13.04 | 93.93 ± 10.67 | **107.86 ± 2.25** |
|  |  | Aperture-restricted | **98.79 ± 0.46** | 110.33 ± 2.33 | 97.02 ± 2.57 | 99.44 ± 1.01 | 108.32 ± 2.39 | **98.27 ± 1.80** | **99.72 ± 0.37** | **108.20 ± 2.17** | **85.69 ± 8.24** | **95.69 ± 4.66** | 108.18 ± 2.15 |

**Table S4.** Paired Wilcoxon signed-rank test results comparing open-field and aperture-restricted field geometries for target coverage metrics across treatment platforms and convergence modes. Statistically significance is indicated by asterisks (**p* < 0.05, ***p* < 0.01, ****p* < 0.001). CW = chest wall; SCV = supraclavicular nodes; AX = axillary nodes; IMN = internal mammary nodes.

| Machine | Convergence mode | PTV_breast/CW | | PTV_SCV | | | PTV_AX | | | PTV_IMN | | |
| --- | --- | --- | --- | --- | --- | --- | --- | --- | --- | --- | --- | --- |
|  |  | V90% (%) | D0.1cc (%) | V95%(%) | V90%(%) | D0.1cc(%) | V95%(%) | V90%(%) | D0.1cc(%) | V95%(%) | V90%(%) | D0.1cc(%) |
| TrueBeam | Off | 0.030* | 0.091 | 0.217 | 0.173 | 0.119 | 0.001** | <0.001*** | 0.009** | 0.426 | 0.626 | 0.217 |
|  | On | 0.135 | 0.426 | 0.017* | 0.078 | 0.903 | <0.001*** | <0.001*** | 0.013* | 0.903 | 0.855 | 0.670 |
|  | Extended | 0.463 | 0.241 | 0.011* | 0.068 | 0.135 | <0.001*** | <0.001*** | 0.013* | 0.296 | 0.241 | 0.761 |
| Halcyon | Off | 0.808 | 0.626 | 0.855 | 0.808 | 0.670 | <0.001*** | 0.001** | 0.042* | 0.670 | 0.761 | 0.952 |
|  | On | 0.855 | 0.049* | 0.761 | 0.542 | 0.119 | <0.001*** | <0.001*** | 0.626 | 0.903 | 0.903 | 0.326 |
|  | Extended | 0.761 | 0.119 | 0.241 | 0.502 | 0.042* | 0.002** | <0.001*** | 0.326 | 0.808 | 0.808 | 0.502 |
| Versa HD | Off | 0.502 | 0.903 | 0.583 | 0.542 | 0.715 | 0.002** | 0.001** | 0.391 | 0.326 | 0.670 | 0.542 |
|  | On | 0.194 | 0.104 | 1.000 | 0.426 | 0.358 | 0.001** | 0.001** | 0.542 | 0.952 | 0.808 | 0.855 |
|  | Extended | 0.426 | 0.135 | 0.715 | 0.670 | 0.426 | 0.001** | <0.001*** | 0.626 | 0.715 | 0.855 | 1.000 |

**Table S5.** Significant Holm-adjusted p-values from pairwise comparisons of convergence mode settings for target coverage and hotspot metrics, shown for each treatment platform and field geometry. Blank cells denote comparisons that were either not tested due to a non-significant Friedman result or not statistically significant after Holm adjustment. Numbers highlighted in Gray indicated a more restrictive convergence mode resulted in significantly worse dosimetric performance. However, the use of “On” and “Extended” convergence modes was associated with significant reductions in hotspot dose, with both modes outperforming “Off” and “Extended” further improving upon “On”. CW = chest wall; SCV = supraclavicular nodes; AX = axillary nodes; IMN = internal mammary nodes.

| Machine | Field geometry | Convergence mode | PTV_breast/CW | | PTV_SCV | | | PTV_AX | | | PTV_IMN | | |
| --- | --- | --- | --- | --- | --- | --- | --- | --- | --- | --- | --- | --- | --- |
|  |  |  | V90% (%) | D0.1cc (%) | V95%(%) | V90%(%) | D0.1cc(%) | V95%(%) | V90%(%) | D0.1cc(%) | V95%(%) | V90%(%) | D0.1cc(%) |
| TrueBeam | Open-field | Off vs On | 0.004 | 0.013 | 0.007 |  | 0.013 |  |  | 0.006 |  |  | 0.010 |
|  |  | Off vs Extended | <0.001 | <0.001 |  |  | <0.001 |  |  | <0.001 |  |  | 0.002 |
|  |  | On vs Extended | 0.001 |  |  |  |  |  |  |  |  |  | 0.030 |
|  | Aperture-restricted | Off vs On | 0.001 | <0.001 |  |  | 0.006 |  |  | <0.001 |  |  | <0.001 |
|  |  | Off vs Extended | 0.001 | <0.001 |  |  | <0.001 |  |  | <0.001 |  |  | <0.001 |
|  |  | On vs Extended | 0.004 | 0.002 |  |  | 0.006 |  |  | 0.006 |  |  | 0.011 |
| Halcyon | Open-field | Off vs On | 0.001 | <0.001 |  |  | <0.001 | 0.041 |  | <0.001 |  |  | <0.001 |
|  |  | Off vs Extended | <0.001 | <0.001 |  |  | <0.001 | 0.026 |  | <0.001 |  |  | <0.001 |
|  |  | On vs Extended | 0.001 | 0.005 |  |  | <0.001 | 0.041 |  | <0.001 |  |  | <0.001 |
|  | Aperture-restricted | Off vs On | <0.001 | 0.003 |  |  | 0.006 |  |  | 0.002 |  | 0.050 | <0.001 |
|  |  | Off vs Extended | <0.001 | <0.001 |  |  | 0.004 |  |  | 0.001 |  | 0.050 | <0.001 |
|  |  | On vs Extended | 0.001 | 0.003 |  |  | 0.025 |  |  |  |  |  | 0.013 |
| Versa HD | Open-field | Off vs On | <0.001 | <0.001 |  |  | <0.001 |  |  | <0.001 |  |  | <0.001 |
|  |  | Off vs Extended | <0.001 | <0.001 |  |  | <0.001 | 0.026 |  | <0.001 |  |  | <0.001 |
|  |  | On vs Extended | 0.025 |  |  |  | <0.001 |  |  | 0.002 |  |  | 0.007 |
|  | Aperture-restricted | Off vs On | 0.002 | 0.013 |  |  | 0.002 |  |  | <0.001 |  | 0.013 | 0.004 |
|  |  | Off vs Extended | 0.002 | <0.001 |  |  | <0.001 |  |  | <0.001 |  | 0.012 | <0.001 |
|  |  | On vs Extended | 0.003 | <0.001 |  |  | <0.001 |  |  | <0.001 |  |  | 0.001 |

**Table S6.** Significant Holm-adjusted p-values from pairwise comparisons of convergence mode settings for heart Dmean, left lung V17Gy, right lung V4Gy, right breast V5Gy and MUs, summarized by treatment platform and field geometry. Blank cells indicate comparisons that were either not evaluated due to a non-significant Friedman test or not statistically significant after Holm adjustment. Where statistically significant differences were observed, the use of “On” and “Extended” convergence settings were associated with reduced OAR dose compared with the default “Off” setting, with the “Extended” mode generally providing additional improvement relative to the “On” mode.

| Machine | Field geometry | Convergence mode | Heart Dmean (Gy) | Left Lung  V17Gy (%) | Right lung  V4Gy (%) | Right breast/CW V5Gy (%) | MUs |
| --- | --- | --- | --- | --- | --- | --- | --- |
| TrueBeam | Open-field | Off vs On | 0.004 | <0.001 | 0.009 |  |  |
|  |  | Off vs Extended | <0.001 | <0.001 | 0.021 |  | 0.027 |
|  |  | On vs Extended | <0.001 | <0.001 |  |  | 0.020 |
|  | Aperture-restricted | Off vs On | <0.001 | <0.001 | <0.001 |  |  |
|  |  | Off vs Extended | <0.001 | <0.001 | 0.002 | 0.001 | 0.010 |
|  |  | On vs Extended | <0.001 | <0.001 | 0.035 | 0.001 | <0.001 |
| Halcyon | Open-field | Off vs On | <0.001 | <0.001 | 0.003 |  |  |
|  |  | Off vs Extended | <0.001 | <0.001 | 0.010 |  |  |
|  |  | On vs Extended | <0.001 | <0.001 | 0.017 |  |  |
|  | Aperture-restricted | Off vs On | <0.001 | <0.001 | 0.010 |  |  |
|  |  | Off vs Extended | <0.001 | <0.001 | 0.003 |  | 0.021 |
|  |  | On vs Extended | <0.001 | 0.009 | 0.042 | 0.001 | 0.003 |
| Versa HD | Open-field | Off vs On | <0.001 | <0.001 | 0.026 |  | 0.005 |
|  |  | Off vs Extended | <0.001 | <0.001 | 0.026 |  | 0.005 |
|  |  | On vs Extended | <0.001 | <0.001 | 0.026 |  |  |
|  | Aperture-restricted | Off vs On | <0.001 | <0.001 |  |  |  |
|  |  | Off vs Extended | <0.001 | <0.001 |  |  |  |
|  |  | On vs Extended | <0.001 | <0.001 | 0.003 |  |  |

**Table S7.** Additional dosimetric metrics (mean ± standard deviation) for the open-field and aperture-restricted VMAT plans across linac platforms and convergence modes, including contralateral breast/CW Dmean and Dmax and metrics corresponding to the suggested additional OAR dose considerations specified in the ASTRO–ASCO–SSO guideline.

| **Machine** | **Convergence mode** | **Field geometry** | **Right breast/CW Dmean (Gy)** | **Right breast/CW Dmax (Gy)** | **Brachial**  **Plexus_L D0.1cc (%)** | **Esophagus V8Gy (%)** | **Esophagus V17Gy (%)** | **Ventricle_L V1.6Gy (%)** | **SpinalCord D0.1cc (Gy)** | **Thyroid Dmean (Gy)** | **Humeral Head Dmean (Gy)** | **Stomach Dmean (Gy)** |
| --- | --- | --- | --- | --- | --- | --- | --- | --- | --- | --- | --- | --- |
| TrueBeam | Off | Open-field | 2.02 ± 0.75 | 16.18 ± 8.14 | 108.40 ± 3.48 | 30.57 ± 15.70 | 7.58 ± 5.80 | 86.80 ± 14.46 | 12.81 ± 2.29 | 14.97 ± 6.83 | 6.97 ± 4.56 | 1.88 ± 1.94 |
|  |  | Aperture-restricted | 2.55 ± 0.89 | 17.06 ± 8.61 | 105.64 ± 2.27 | 24.92 ± 17.79 | 8.09 ± 5.19 | 74.29 ± 18.17 | 13.56 ± 4.69 | 14.38 ± 6.45 | 5.95 ± 3.50 | 1.04 ± 0.68 |
|  | On | Open-field | 1.94 ± 0.89 | 18.89 ± 8.82 | 106.47 ± 3.85 | 25.86 ± 15.71 | 6.99 ± 5.49 | 82.45 ± 17.45 | 12.06 ± 3.28 | 15.85 ± 6.70 | 7.65 ± 4.74 | 1.47 ± 1.44 |
|  |  | Aperture-restricted | 2.32 ± 1.00 | 18.39 ± 8.97 | 104.06 ± 2.35 | 22.47 ± 15.61 | 6.84 ± 5.31 | 71.06 ± 19.78 | 13.03 ± 5.22 | 14.30 ± 6.38 | 6.14 ± 3.57 | 1.00 ± 0.65 |
|  | Extended | Open-field | 1.93 ± 0.96 | 19.23 ± 9.96 | 105.98 ± 3.48 | 25.42 ± 15.93 | 6.89 ± 6.20 | 80.79 ± 19.32 | 12.07 ± 3.85 | 16.24 ± 7.00 | 7.78 ± 4.67 | 1.35 ± 1.26 |
|  |  | Aperture-restricted | 2.07 ± 0.91 | 19.60 ± 8.79 | 103.51 ± 2.37 | 21.74 ± 13.60 | 6.20 ± 5.65 | 68.80 ± 20.92 | 12.69 ± 4.72 | 13.89 ± 6.25 | 6.31 ± 3.89 | 1.01 ± 0.69 |
| Halcyon | Off | Open-field | 2.03 ± 0.78 | 13.88 ± 7.05 | 105.51 ± 2.71 | 29.19 ± 13.90 | 7.00 ± 5.92 | 75.35 ± 18.39 | 13.84 ± 3.91 | 13.92 ± 6.22 | 6.42 ± 4.04 | 1.59 ± 1.58 |
|  |  | Aperture-restricted | 2.57 ± 0.87 | 15.49 ± 7.43 | 104.71 ± 2.37 | 23.58 ± 15.50 | 7.50 ± 4.25 | 71.77 ± 17.63 | 13.88 ± 4.92 | 14.00 ± 5.95 | 5.93 ± 3.60 | 1.20 ± 0.85 |
|  | On | Open-field | 2.02 ± 0.91 | 16.15 ± 8.42 | 103.59 ± 2.48 | 24.78 ± 13.74 | 6.41 ± 5.64 | 71.00 ± 19.24 | 13.19 ± 4.48 | 14.23 ± 5.92 | 6.76 ± 4.26 | 1.42 ± 1.26 |
|  |  | Aperture-restricted | 2.40 ± 1.01 | 16.88 ± 7.86 | 103.50 ± 2.47 | 22.55 ± 15.26 | 7.25 ± 4.85 | 68.55 ± 19.11 | 13.39 ± 5.04 | 14.14 ± 5.91 | 5.88 ± 3.50 | 1.14 ± 0.75 |
|  | Extended | Open-field | 1.85 ± 0.85 | 18.37 ± 9.64 | 102.20 ± 2.31 | 23.62 ± 13.81 | 6.38 ± 5.83 | 67.09 ± 20.46 | 12.91 ± 4.46 | 14.34 ± 5.98 | 6.75 ± 4.07 | 1.22 ± 0.95 |
|  |  | Aperture-restricted | 2.14 ± 0.88 | 19.57 ± 9.33 | 102.83 ± 2.18 | 22.98 ± 17.49 | 6.56 ± 5.10 | 66.30 ± 19.84 | 13.28 ± 5.40 | 13.83 ± 5.68 | 5.91 ± 3.36 | 1.07 ± 0.70 |
| Versa HD | Off | Open-field | 2.13 ± 0.73 | 13.66 ± 6.85 | 106.90 ± 3.46 | 30.56 ± 16.81 | 6.77 ± 5.65 | 83.81 ± 15.04 | 13.75 ± 3.76 | 14.09 ± 6.73 | 6.56 ± 4.56 | 1.75 ± 1.51 |
|  |  | Aperture-restricted | 2.91 ± 0.98 | 16.97 ± 7.31 | 106.40 ± 2.36 | 24.96 ± 16.14 | 8.10 ± 4.72 | 79.44 ± 13.90 | 13.84 ± 4.66 | 14.70 ± 6.32 | 5.83 ± 3.62 | 1.24 ± 0.83 |
|  | On | Open-field | 2.12 ± 0.79 | 14.47 ± 7.89 | 105.09 ± 3.05 | 26.26 ± 13.82 | 6.38 ± 5.42 | 81.85 ± 16.12 | 13.34 ± 4.18 | 14.12 ± 6.26 | 6.74 ± 4.72 | 1.49 ± 1.08 |
|  |  | Aperture-restricted | 2.82 ± 1.00 | 17.39 ± 8.11 | 105.07 ± 2.32 | 24.26 ± 16.56 | 7.67 ± 4.77 | 78.70 ± 14.67 | 13.46 ± 5.08 | 14.55 ± 6.06 | 5.91 ± 3.61 | 1.17 ± 0.70 |
|  | Extended | Open-field | 2.08 ± 0.89 | 15.14 ± 8.10 | 104.61 ± 2.81 | 24.97 ± 14.26 | 6.14 ± 5.23 | 80.26 ± 16.67 | 13.19 ± 4.41 | 14.37 ± 6.16 | 6.72 ± 4.55 | 1.37 ± 0.91 |
|  |  | Aperture-restricted | 2.67 ± 0.98 | 17.80 ± 8.51 | 104.34 ± 2.27 | 23.21 ± 14.01 | 7.33 ± 4.51 | 77.73 ± 15.15 | 13.17 ± 4.80 | 14.62 ± 6.11 | 5.98 ± 3.91 | 1.12 ± 0.63 |

**Table S8**. Post-hoc power analysis and 95% confidence intervals (CI) for the mean heart dose reduction associated with the aperture-restricted geometry. To further evaluate the sample size, the mean heart dose reduction was averaged across the three treatment delivery platforms within each convergence mode while preserving the patient as the unit of analysis.

| **Convergence mode** | **Open-field (Gy)** | **Aperture-restricted (Gy)** | **Mean reduction (Gy)** | **SD of difference (Gy)** | **95% CI of mean reduction (Gy)** | **Cohen's *d*z** | **Achieved power** |
| --- | --- | --- | --- | --- | --- | --- | --- |
| Off | 3.829 | 2.711 | 1.118 | 1.204 | 0.423 to 1.813 | 0.929 | 89.4% |
| On | 3.251 | 2.526 | 0.725 | 0.796 | 0.265 to 1.185 | 0.911 | 88.2% |
| Extended | 2.966 | 2.371 | 0.595 | 0.640 | 0.226 to 0.965 | 0.930 | 89.5% |
