# Supplementary material for: Dosimetric impact of aperture‐restricted VMAT on heart dose in breast cancer treated with regional nodal irradiation
Source: J Appl Clin Med Phys. 2026 Aug 26;27(9):e70775. doi: 10.1002/acm2.70775 (PMC13507928; doi:10.1002/acm2.70775)
Supplement: Supplementary file 2 [file ACM2-27-e70775-s002.docx]

**Figure S1.**  Beam’s-eye view (BEV) of a representative case at the control point corresponding to gantry 315°, illustrating the three linac and collimator configurations: (A) Varian TrueBeam, (B) Varian Halcyon, and (C) Elekta Versa HD. Contour legends are shown in (A).

**Figure S2.** Flash: (A) A 1 cm virtual bolus (HU = −500; blue contour) is placed over the PTV breast. An optimization structure is then generated by expanding the PTV breast (orange contour) by 1 cm anteriorly and laterally (purple contour). (B) Representative BEV from the patient’s left lateral showing MLC aperture with flash after the bolus is removed.

**Figure S3.** Illustrative examples of the potential geometric explanation for the increased contralateral breast/CW dose observed with the aperture-restricted beam geometry. Restricting the deep field apertures reduces the available beam directions for covering the medial portion of the PTV. To maintain target coverage, a greater dose contribution from the remaining beam directions, particularly left-to-right directions, may result in additional exit dose through the contralateral breast/CW. The top example (A) shows a representative patient with an intact contralateral breast, and the bottom example (B) shows a representative patient who underwent bilateral mastectomy.
